# Supplementary material for: Effect of Siponimod on Brain and Spinal Cord Imaging Markers of Neurodegeneration in the Theiler’s Murine Encephalomyelitis Virus Model of Demyelination
Source: Int J Mol Sci. 2023 Aug 20;24(16):12990. doi: 10.3390/ijms241612990 (PMC10455446; doi:10.3390/ijms241612990)
Supplement: Supplementary file 1 [file ijms-24-12990-s001.zip › ijms-2504917-supplementary.pdf]

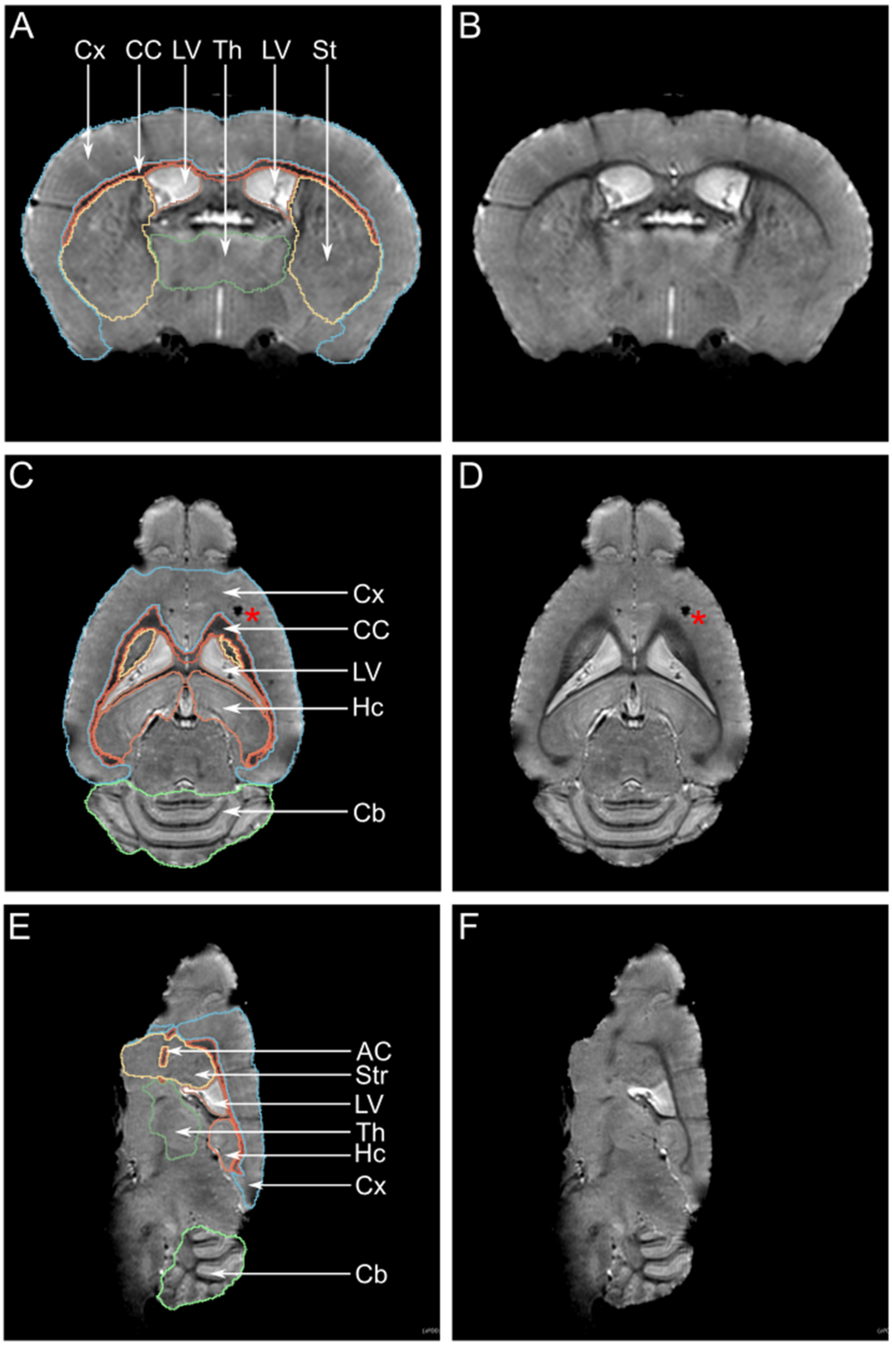

Supplementary Figure S1. Atlas based segmentation of mouse brain scans images: The figure presents representative results from automated segmentation pipeline used for quantifying region specific volumes in each brain scan image. **A** and **B** present coronal section presenting a coronal view for a mouse brain scan with and without region segmentation label outlines respectively. **C** and **D** present coronal section presenting a sagittal view for a mouse brain scan with and without region segmentation label outlines respectively. Red asterix * in these panel signal from TMEV IC injection site. **E** and **F** present axial section presenting a coronal view for a

mouse brain scan with and without region segmentation label outlines respectively. Arrows labeled as AC, Cb, CC, Cx, Hc, LV, St and Th mark anterior commissure, cerebellum, corpus callosum, iso-cortex, hippo campus, lateral ventricles, striatum and thalamus respectively

Supplement Table S1:- Table S1 rows specify the number of animals in each treatment group. Table S1 columns specify the number of animals in each scan protocol from each treatment group.

	**Spine Scan**	**Brain Scan**	**No Scan**	**Total**
**Siponimod Treated**	7	15	22	44
**Vehicle Treated**	7	15	22	44
**Healthy Control**	3	5	-	8
**Sham Control**	-	6	-	6
